# Foxp2 loss of function increases striatal direct pathway inhibition via increased GABA release

**DOI:** 10.1007/s00429-018-1746-6

**Published:** 2018-09-05

**Authors:** Jon-Ruben van Rhijn, Simon E. Fisher, Sonja C. Vernes, Nael Nadif Kasri

**Affiliations:** 1 Department of Cognitive Neuroscience, Donders Institute for Brain, Cognition and Behaviour, Radboudumc, 6525 HR Nijmegen, The Netherlands; 2 Neurogenetics of Vocal Communication Group, Max Planck Institute for Psycholinguistics, 6525 XD Nijmegen, The Netherlands; 3 Language and Genetics Department, Max Planck Institute for Psycholinguistics, 6525 XD Nijmegen, The Netherlands; 4 Donders Institute for Brain, Cognition and Behaviour, 6525 HR Nijmegen, The Netherlands; 5 Department of Human Genetics, Donders Institute for Brain, Cognition, and Behaviour, Radboudumc, 6525 GA Nijmegen, The Netherlands

**Keywords:** Foxp2, Excitatory-inhibitory balance, Neurodevelopment, D1R-MSN, Striatum

## Abstract

Heterozygous mutations of the Forkhead-box protein 2 (*FOXP2*) gene in humans cause childhood apraxia of speech. Loss of Foxp2 in mice is known to affect striatal development and impair motor skills. However, it is unknown if striatal excitatory/inhibitory balance is affected during development and if the imbalance persists into adulthood. We investigated the effect of reduced Foxp2 expression, via a loss-of-function mutation, on striatal medium spiny neurons (MSNs). Our data show that heterozygous loss of Foxp2 decreases excitatory (AMPA receptor-mediated) and increases inhibitory (GABA receptor-mediated) currents in D1 dopamine receptor positive MSNs of juvenile and adult mice. Furthermore, reduced Foxp2 expression increases GAD67 expression, leading to both increased presynaptic content and release of GABA. Finally, pharmacological blockade of inhibitory activity in vivo partially rescues motor skill learning deficits in heterozygous *Foxp2* mice. Our results suggest a novel role for Foxp2 in the regulation of striatal direct pathway activity through managing inhibitory drive.

**Electronic supplementary material:**

The online version of this article (10.1007/s00429-018-1746-6) contains supplementary material, which is available to authorized users.

## Introduction

Balanced neuronal activity between cortex, striatum and thalamus is essential for the generation of voluntary movements (Shepherd 2013). Imbalanced activity within the striatum is known to disrupt complex motor behaviors, such as the production of spoken language (Peach 2004; Square-Storer et al. 1990). *FOXP2*, the first single gene linked to a speech and language disorder (Lai et al. 2001), is important for the correct execution of complex motor behaviors used for speech. Individuals with mutations in the *FOXP2* gene have problems executing coordinated sequences of orofacial movements, which impede their speech [diagnosed as developmental verbal dyspraxia or childhood apraxia of speech (CAS)], while their general cognitive functioning and other aspects of motor coordination are usually less severely affected (MacDermot et al. 2005; Morgan et al. 2017). Mice with heterozygous Foxp2 mutations display impairments in motor skill learning, shown by decreased performance on the accelerating rotarod (French et al. 2012; Groszer et al. 2008), suggesting that similar neurobiological substrates could underlie the behavioral phenotypes in human and mouse. *FoxP2* codes for a transcription factor (Devanna et al. 2014; Vernes et al. 2006, 2007) and plays important roles during the early development of the central nervous system as well as in the postnatal brain (Spiteri et al. 2007; Vernes et al. 2011; Groszer et al. 2008). Mutations of this gene affect both cortical and striatal activity in human cases and animal models (French et al. 2012; Groszer et al. 2008; Liegeois et al. 2003). Of particular note, striatal long-term depression is affected in adult mice with heterozygous *Foxp2* mutations (Groszer et al. 2008; Enard et al. 2009), which suggests that Foxp2 regulates molecular mechanisms involved in synaptic plasticity. Additionally, evidence from in vivo recordings shows that *Foxp2* mutant mice display abnormal ongoing striatal activity and dysregulated firing rates during a motor-learning task (French et al. 2012). Finally, Foxp2 has been reported to regulate genes involved in synapse formation (Sia et al. 2013; Vernes et al. 2011) and was recently shown to affect excitatory synaptic activity during early postnatal development through inhibition of the *Mef2c* gene (Chen et al. 2016).

Studies using mouse models to investigate the functions of Foxp2 have made use of two well described mutations which differentially affect Foxp2 and are similar to mutations described in patients with CAS. These mutations lead to either disruption of the DNA binding domain of Foxp2, or a loss of function stop-gain mutation in exon 7 that causes nonsense mediated decay of Foxp2 protein (MacDermot et al. 2005; Morgan et al. 2017). Though neurobiological mechanisms affected by these different mutations could differ, there is currently no data to suggest this. Moreover, heterozygous Foxp2 mice with either the DNA binding domain mutation or the loss of function mutation display similar impairments in motor skill learning (French et al. 2012; Enard et al. 2009; Groszer et al. 2008).

To date, investigations into the functions of Foxp2 in striatum have focused on how Foxp2 affects excitatory activity (Groszer et al. 2008; Enard et al. 2009; French et al. 2012; Chen et al. 2016; Schreiweis et al. 2014). Although the striatum receives numerous excitatory connections from the cortex (Shepherd 2013) and thalamus (Smith et al. 2004, 2009), it is itself entirely composed of inhibitory neurons (Kreitzer and Malenka 2008). GABAergic medium spiny neurons (MSNs) make up 95% of the striatum, and two major populations can be distinguished: MSNs that express either the D1 dopamine receptor (D1R-MSNs) or the D2 dopamine receptor (D2R-MSNs) (Gittis and Kreitzer 2012). These MSN populations differentially affect the downstream neural sites to which they ultimately project, and each regulate separate aspects of motor behavior (Calabresi et al. 2014; Surmeier et al. 2007; Gittis and Kreitzer 2012). D1R-MSNs innervate the direct pathway, which leads to increased activation of the cortico-striatal-thalamic motor circuit. In contrast, D2R-MSNs belong to the indirect pathway, inactivating this motor circuit. Balanced excitation and inhibition (E/I balance) of cells within both striatal pathways is crucial for the generation of complex motor behaviors (Schroll et al. 2015).

How Foxp2 affects neuronal function has been investigated in both early development and adulthood, but knowledge of how Foxp2 affects striatal circuits during (motor) development is lacking. This is especially important to address since E/I balance is dynamic. Changes in E/I balance during development are tightly regulated and have been described in multiple cell types in hippocampus (Liu 2004) and cortex (Zhang et al. 2011) of juvenile mice. A disrupted E/I balance during development can severely affect adult behavior (Peixoto et al. 2016). Indeed, aberrant E/I balance in striatal cells is known to lead to impaired motor learning in adult mice (Rothwell et al. 2014), similar to the deficits observed in adult mice with mutations in *Foxp2* (French et al. 2012; Groszer et al. 2008).

We examined the effects of reduced Foxp2 expression from early development into adulthood in the striatum, using a heterozygous mouse model for the stop-gain Foxp2 mutation (S321X). Foxp2 protein expression is absent in *Foxp2*^*S321X*/*S321X*^ mice and reduced to intermediate levels in *Foxp2*^*S321X*/+^mice (Groszer et al. 2008; Vernes et al. 2011). We provide evidence that Foxp2 plays a role in the regulation of striatal E/I balance, regulates inhibitory activity through repression of GAD67, and regulates inhibitory presynaptic strength of D1R-MSNs, but not D2R-MSNs. Finally, we show that pharmacological blockade of striatal inhibition partially rescues the motor skill learning deficits observed in heterozygous *Foxp2* mutant mice. Taken together, our results reveal a developmental circuit defect caused by reduced levels of functional Foxp2, which suggests that E/I imbalances in striatal activity may contribute to (vocal) motor problems in humans with *FOXP2* mutations.

## Results

### Reduced Foxp2 expression affects D1R-MSN excitatory synaptic inputs

**Fig. 1:**
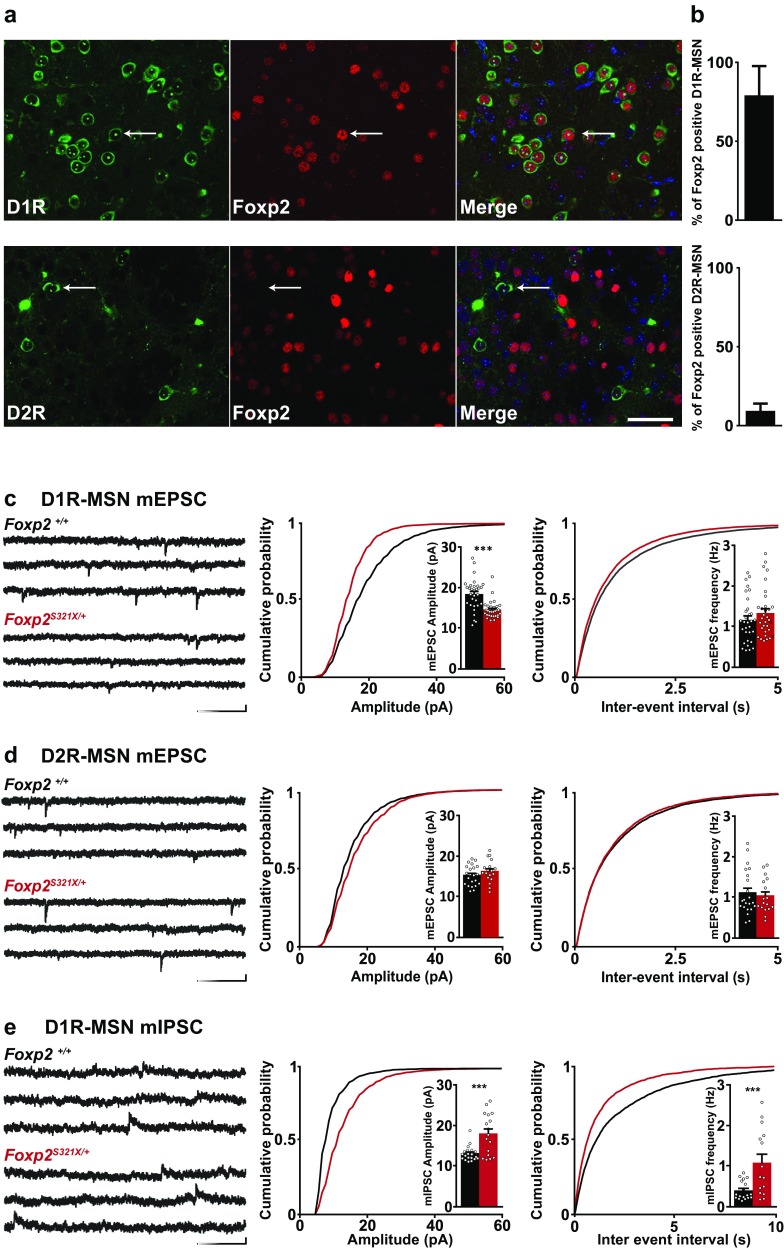
Foxp2 is predominantly expressed in D1R-MSNs in dorsolateral striatum and affects synaptic activity. **a** D1R-GFP, D2R-GFP and Foxp2 are expressed in a subset of striatal cells in juvenile (PND11-14) mice, arrows point to cells with overlapping D1R and Foxp2 expression (top row) or D2R expression without Foxp2 expression (bottom row). Scale bar 50 µm. **b** 83.7 ± 16% of D1R-GFP positive cells express Foxp2, compared to 16.9 ± 4% of D2R-GFP cells. *N*/*n* number of mice/number of slices. *N*/*n* = 3/12. **c** Striatal D1R-MSN mEPSC amplitude is decreased following reduced Foxp2 expression. Example of mEPSC activity in striatal D1R-MSNs from juvenile (PND14) *Foxp2*^+/+^ and *Foxp2*^*S321X*/+^ mice. Scale bar 200 ms/20 pA. Cumulative distribution of mEPSC amplitude (*Foxp2*^+/+^ = 19.4 ± 0.69 pA, *Foxp2*^*S321X*/+^ = 14.3 ± 0.36 pA, *P* < 0.0001) and frequency (*Foxp2*^+/+^ = 1.12 ± 0.1 Hz, *Foxp2*^*S321X*/+^ = 1.19 ± 0.11 Hz, NS) in striatal D1R-MSNs. *Foxp2*^+/+^ *N*/*n* = 3/31, *Foxp2*^*S321X*/+^ *N*/*n* = 3/32. **d** Example traces of mEPSC activity in striatal D2R-MSNs from juvenile (PND14) *Foxp2*^+/+^ and *Foxp2*^*S321X*/+^ mice Scale bar 200 ms/20 pA. Cumulative distribution of mEPSC amplitude (*Foxp2*^+/+^ = 15.64 ± 0.53 pA, *Foxp2*^*S321X*/+^ = 16.14 ± 0.62 pA, NS) and frequency (*Foxp2*^+/+^ = 0.92 ± 0.12 Hz, *Foxp2*^*S321X*/+^ = 0.89 ± 0.1 Hz, NS) in striatal D2R-MSNs. *Foxp2*^+/+^ *N*/*n* = 3/22, *Foxp2*^*S321X*/+^ *N*/*n* = 3/19. **e** Example traces of mIPSC activity in striatal D1R-MSNs from juvenile (PND14) *Foxp2*^+/+^ and *Foxp2*^*S321X*/+^ mice. Scale bar 200 ms/10 pA. Cumulative distribution of mIPSC amplitude (*Foxp2*^+/+^ = 8.6 ± 0.27 pA, *Foxp2*^*S321X*/+^ = 11.9 ± 0.83 pA, *P* < 0.001) and frequency (*Foxp2*^+/+^ = 0.18 ± 0.036 Hz, *Foxp2*^*S321X*/+^ = 0.71 ± 0.14 Hz, *P* < 0.01) in striatal D1R-MSNs. *Foxp2*^+/+^ *N*/*n* = 3/20, *Foxp2*^*S321X*/+^ *N*/*n* = 3/17. ***P < 0.01. *N* number of mice, *n* number of cells. All data analyzed by two-sided Students’ *T* test

Previous studies have suggested that Foxp2 is differentially expressed in D1R- versus D2R-expressing MSNs in the striatum (Vernes et al. 2011). To directly assess the expression of Foxp2 in D1R- and D2R-MSNs, we performed immunocytochemistry for Foxp2 on mice containing bacterial artificial chromosome (BAC)-TRAP GFP constructs (Heiman et al. 2008; Doyle et al. 2008) under the D1R or D2R promoter, which have been shown to faithfully label D1R- or D2R-expressing MSNs, respectively (Heiman et al. 2008). Upon investigation of expression in juvenile mice (PND11-14) we found that Foxp2 is expressed in nearly all striatal D1R-MSNs, in contrast to only a small fraction of D2R-MSN (Fig. 1a).

We next investigated whether heterozygous *Foxp2* loss of function differentially affects striatal MSN subtypes at the synaptic level. To enable a targeted single-cell characterization of how reduced Foxp2 expression affects striatal activity, we crossed *Foxp2*^*S321X*/+^ mice with (BAC)-TRAP D1R-GFP mice. We performed patch-clamp recordings on GFP-positive D1R-MSNs and non-GFP putative D2R-MSNs. Although Foxp2 expressing MSNs are spread throughout the striatum, we chose to focus on cells in the dorsolateral striatum, because of its connection to the motor cortex (Hunnicutt et al. 2016). Moreover, previous experiments regarding Foxp2 function have shown aberrant activity in dorsal striatum of heterozygous Foxp2 mutant/knockout mice (French et al. 2012; Groszer et al. 2008; Reimers-Kipping et al. 2011), and reduced motor skill learning suggests areas involved in motor control might be more severely affected by reduced Foxp2 expression. We measured excitatory synaptic strength through glutamatergic α-amino-3-hydroxy-5-methyl-4-isoxazolepropionic acid (AMPA) receptor activation by analysis of miniature excitatory postsynaptic current (mEPSC) amplitude and frequency. In D1R-MSNs of heterozygous postnatal day (PND) 10–14 juvenile mice, mEPSC amplitude was reduced, whereas mEPSC frequency was similar between genotypes (Fig. 1c). No changes in D2R-MSN amplitude or frequency were observed (Fig. 1d). Finally, we measured AMPA/NMDA ratio in D1R-MSNs of juvenile *Foxp2*^+/+^ and *Foxp2*^*S32X*/+^ mice. AMPA/NMDA ratio is significantly increased in *Foxp2*^*S32X*/+^ mice, which suggests that NMDA currents are decreased as well in addition to the previously observed reduction in AMPAR-mediated activity (Suppl Fig. 1). These results show that reduced Foxp2 expression leads to decreased excitatory postsynaptic strength of only direct pathway MSNs, which is consistent with the predominant expression of Foxp2 in D1R-MSNs.

### Inhibitory synaptic inputs are increased in D1R-MSNs of *Foxp2*^*S321X*/+^ mice

Physiological effects of heterozygous Foxp2 mutations have only been investigated in the context of excitatory synaptic transmission (Chen et al. 2016; Reimers-Kipping et al. 2011; Schreiweis et al. 2014). Since E/I balance is important for the development and maintenance of neuronal circuitry, we examined the role of Foxp2 in striatal inhibition. Striatal inhibition is accomplished through extra-striatal as well as intra-striatal sources. From the cortex, GABAergic interneurons project to the striatum and provide inhibitory input (Melzer et al. 2017). However, corticostriatal GABAergic interneurons do not express Foxp2 (Chiu et al. 2014; Hisaoka et al. 2010). We, therefore, expect differences in inhibitory activity between wild-type and *Foxp2*^*S321X*/+^ mice through changes in intra-striatal inhibition, which is regulated through MSNs and striatal interneurons (Taverna et al. 2008; Lalchandani and Vicini 2013). We measured inhibitory activity only in D1R-MSNs, since unidirectional connections between D1R-MSNs are common, while connections between D1R-MSNs and D2R-MSNs are rare (6%) (Taverna et al. 2008). Though D2R-MSNs synapse on D1R-MSNs (27%) the lack of Foxp2 expression in D2R-MSNs, combined with the lack of an excitatory phenotype, suggests that D2R-MSNs cannot be cell-autonomously affected by Foxp2.

We measured miniature inhibitory postsynaptic currents (mIPSCs), which are mediated by GABA and reflect inhibitory synaptic strength. In D1R-MSNs of juvenile (PND10-14) *Foxp2*^*S321X*/+^ mice, we found that mIPSC amplitude and frequency were increased compared to wild-type controls (Fig. 1e). Our data show that reduced Foxp2 expression differentially affects excitatory and inhibitory synaptic strength. There is no compensation for the decreased excitatory activity, but rather this is aggravated by increased inhibition.

### E/I imbalance persists in dorsolateral striatum of adult *Foxp2*^*S321X*/+^ mice

**Fig. 2:**
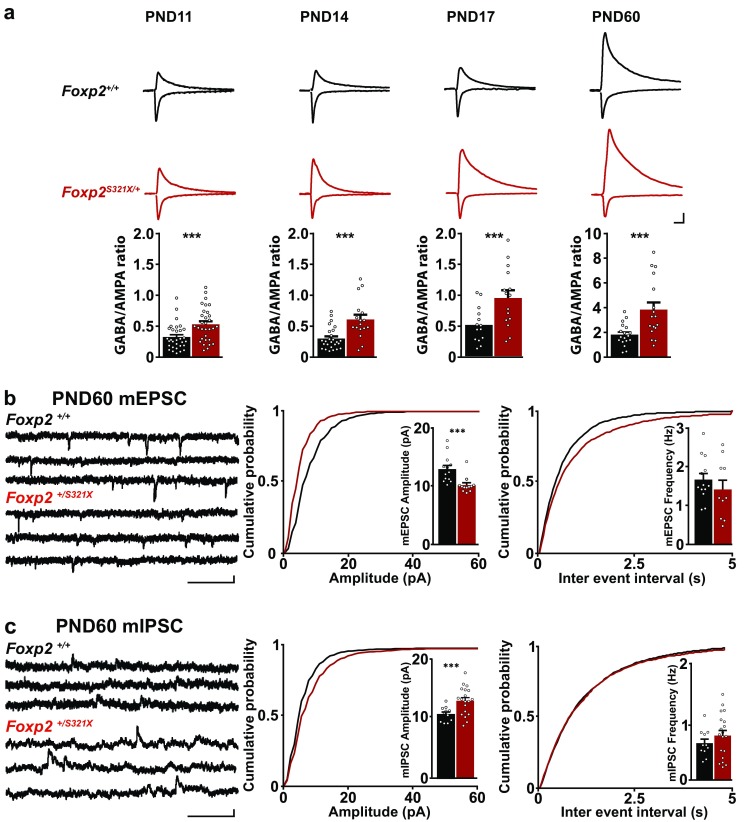
Decreased excitation and increased inhibition persist in adult mice with decreased Foxp2 expression. **a** Example traces show AMPA response (negative) and GABA response (positive) in D1R-MSNs of *Foxp2*^+/+^ and *Foxp2*^*S321X*/+^ mice during development and in adulthood. Scale bar 200/50 pA. GABA/AMPA ratio in D1R-MSNs of *Foxp2*^+/+^ (PND11 = 0.32 ± 0.037, PND14 = 0.29 ± 0.035, PND17 = 0.50 ± 0.077, PND60 = 2.04 ± 0.47) and *Foxp2*^*S321X*/+^ (*Foxp2*^*S321X*/+^ PND11 = 0.53 ± 0.049, PND14 = 0.6 ± 0.078, PND17 = 0.94 ± 0.12, PND60 = 4.5 ± 0.85), mice during development and in adulthood (2-factor ANOVA (genotype × age) = *P* < 0.001 for both factors). *N* number of mice, *n* number of cells. *Foxp2*^+/+^ *N*/*n* = 3/31 (PND11), 3/26 (PND13), 3/14 (PND17), 3/18 (PND60), *Foxp2*^*S321X*/+^ *N*/*n* = 3/31 (PND11), 3/26 (PND13), 3/15 (PND17), 3/17 (PND60). **b** Example traces of mEPSC activity in striatal D1R-MSNs from adult (PND60) *Foxp2*^+/+^ and *Foxp2*^*S321X*/+^ mice. Cumulative distribution of mEPSC amplitude (*Foxp2*^+/+^ = 12.9 ± 0.64 pA, *Foxp2*^*S321X*/+^ = 10.2 ± 0.4 pA, *P* < 0.01, two-sided Student’s *T* test) and frequency (*Foxp2*^+/+^ = 1.68 ± 0.16 Hz, *Foxp2*^*S321X*/+^ = 1.42 ± 0.2 Hz, NS, two-sided Student’s *T* test) in striatal D1R-MSNs. *Foxp2*^+/+^ *N*/*n* = 3/13, *Foxp2*^*S321X*/+^ *N*/*n* = 3/12. **c** Example traces of mIPSC activity in striatal D1R-MSNs from adult (PND60) *Foxp2*^+/+^ and *Foxp2*^*S321X*/+^ mice. Cumulative distribution of mIPSC amplitude (*Foxp2*^+/+^ = 11.36 ± 0.41 pA, *Foxp2*^*S321X*/+^ = 12.9 ± 0.82 pA, *P* < 0.01, two-sided Student’s *T* test) and frequency (*Foxp2*^+/+^ = 0.67 ± 0.07 Hz, *Foxp2*^*S321X*/+^ = 0.81 ± 0.09 Hz, NS, two-sided Student’s *T* test) in striatal D1R-MSNs. *Foxp2*^+/+^ *N*/*n* = 4/11, *Foxp2*^*S321X*/+^ *N*/*n* = 6/19. Scale bar in **b, c** 200 ms/10 pA. ***P < 0.001

In mice, Foxp2 is present during the entire lifespan (Ferland et al. 2003), and expression does not change strongly between juvenile and adult animals (Ferland et al. 2003; Takahashi et al. 2003). However, given that this gene is important for early neuronal development (Vernes et al. 2011; Chen et al. 2016), it is conceivable that functional effects of reduced Foxp2 expression differ between juvenile and adult animals. In previous studies the effects of *Foxp2* mutations on striatal physiology have only been investigated in adult (French et al. 2012; Groszer et al. 2008) or juvenile mice separately (Chen et al. 2016), and thus a developmental profile of synaptic changes due to reduced Foxp2 expression is lacking. We hypothesized that the E/I imbalance present in D1R-MSNs of juvenile *Foxp2*^*S321X*/+^ mice might persist until adulthood, since adult *Foxp2* heterozygous mice show clear deficits in motor skill learning. We measured the GABA/AMPA ratio as an index of E/I balance in juvenile PND11, PND14, PND17 as well as adult (PND60) mice, which comprises a developmental profile at ages around the critical time points for the emergence of motor coordination (Dehorter et al. 2011) and striatal synaptic integration and circuit formation in mice (Lee and Sawatari 2011). During development in wild type mice, the GABA/AMPA ratio increases sharply in D1R-MSNs (Fig. 2a). Interestingly, both during development and in adulthood the GABA/AMPA ratio of D1R-MSNs was significantly higher in *Foxp2*^*S321X*/+^ mice than in wild-type controls (Fig. 2a), which indicates that the E/I imbalance we uncovered in juvenile mice indeed persists into adulthood. We subsequently measured mEPSCs and mIPSCs in D1R-MSNs of adult (PND60) *Foxp2*^*S321X*/+^ mice to determine if the increased GABA/AMPA ratios in *Foxp2*^*S321X*/+^ mice reflect persistent changes in excitatory and/or inhibitory synaptic strength. Our results show that the increased GABA/AMPA ratio in adult heterozygous mice is due to decreased mEPSC amplitude (Fig. 2b) coupled with an increased mIPSC amplitude (Fig. 2c). However, the increased mIPSC frequency we had observed in our juvenile mice was not present in adult mice (Fig. 2c), which indicates that some form of compensation might be present. This compensation is, however, insufficient to return activity to baseline levels and, therefore, we conclude that the changes in E/I balance are persistent into adulthood. Changes in inhibitory synaptic strength can indicate changes at either the pre- or the post-synapse, such as increased presynaptic neurotransmitter release or increased expression of postsynaptic GABA receptors, respectively. We, therefore, set out to assess the effect of reduced Foxp2 expression on striatal synapses at the molecular level.

### Decreased Foxp2 expression leads to increased GAD67 expression around D1R-MSN somata

Foxp2 might modulate inhibitory activity by transcriptionally regulating genes involved in GABA signaling (Vernes et al. 2007, 2011; Fujita et al. 2008). One target gene identified in an in vivo chromatin immunoprecipitation (ChIP)-chip screen for Foxp2 binding in mouse brain was *Gad* (Vernes et al. 2011), the gene that codes for GAD67, a key enzyme in the production of GABA at the synapse (Lau and Murthy 2012). By contrast, other genes involved in GABAergic activity, such as VGAT or GAD2, were not detected in this ChIP screen. Moreover, GAD2 expression has been shown to be unaltered in striatal tissue from Foxp2 heterozygous knockout embryos (French et al. 2007). Based on these findings we hypothesized that reduced Foxp2 expression could lead to changes in GAD67 expression, and thus contribute to aberrant GABAergic activity.

**Fig. 3:**
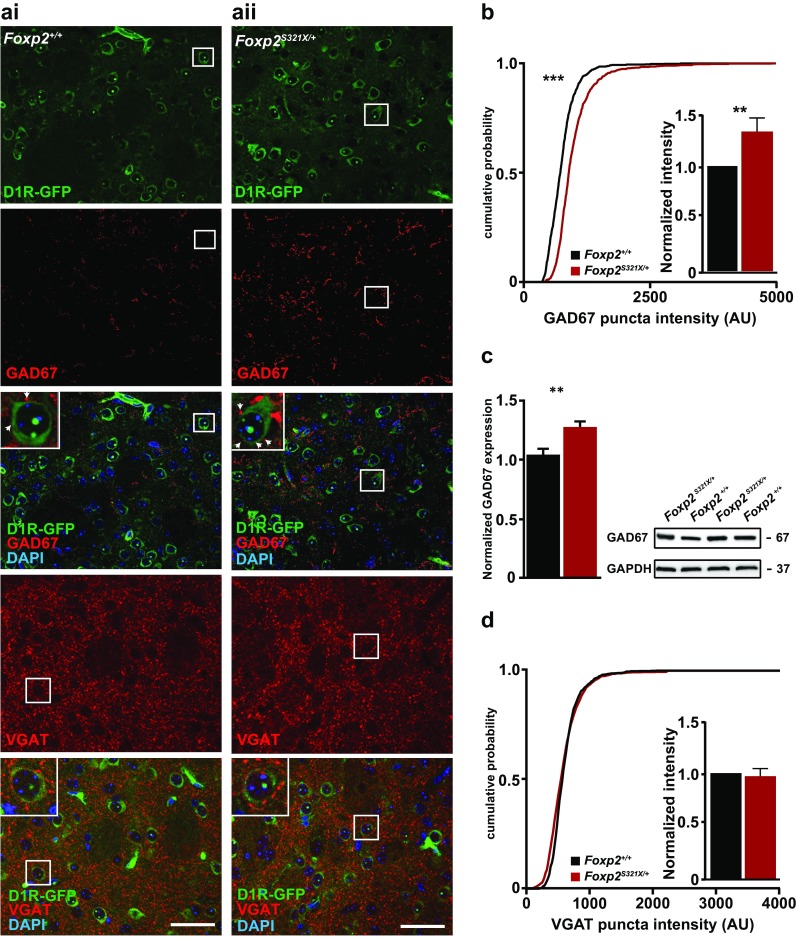
Increased GAD67 expression in *Foxp2*^*S321X*/+^ mice (**a**i, **a**ii). Overview of GAD67 and VGAT expression levels in striatal slices of juvenile (PND10-14) *Foxp2*^+/+^ and *Foxp2*^*S321X*/+^ mice. Insets show puncta which surround D1R-MSN somata. These perisomatic puncta were used for intensity analysis, to restrict analysis to D1R-MSNs. **b** Comparison of GAD67 expression (both cumulative distribution in arbitrary units (AU) and normalized expression) around D1R-GFP positive somata in dorsolateral striatum of juvenile (PND10-14) *Foxp2*^+/+^ and *Foxp2*^*S321X*/+^ mice. All data were compared to normalized expression levels in *Foxp2*^+/+^ mice. (*Foxp2*^*S321X*/+^ 1.32 ± 0.12, *P* < 0.01, Mann–Whitney *U*). Kolmogorov–Smirnov (KS) test was used for the cumulative distribution data, *P* < 0.001. *Foxp2*^+/+^ *N*/*n* = 5/36, *Foxp2*^*S321X*/+^ *N*/*n* = 5/36. **c** Quantification and representative western blot of GAD67 protein expression in juvenile *Foxp2*^+/+^ and *Foxp2*^*S321X*/+^ mice (*Foxp2*^+/+^ 1.04 ± 0.05, *Foxp2*^*S321X*/+^ 1.28 ± 0.04, *P* < *0.01*, two-sided Student’s *T* test. *Foxp2*^+/+^ and *Foxp2*^*S321X*/+^ *N* = 6). **d** Comparison of VGAT expression [both cumulative distribution in arbitrary units (AU) and normalized expression] around D1R-GFP positive somata in dorsolateral striatum of juvenile (PND10-14) *Foxp2*^+/+^ and *Foxp2*^*S321X*/+^ mice. *Foxp2*^*S321X*/+^ 0.97 ± 0.07, NS, Mann–Whitney *U*. KS test for cumulative distribution data: NS. *Foxp2*^+/+^ *N*/*n* = 3/12, *Foxp2*^*S321X*/+^ *N*/*n* = 3/12. *N*/*n*: number of animals/number of slices *P < 0.05, **P < 0.01, ***P < 0.001

We compared GAD67 expression around D1R-MSN somata in the striatum of juvenile *Foxp2*^+/+^ and *Foxp2*^*S321X*/+^ mice (Fig. 3a). GAD67 puncta surrounding striatal D1R-MSNs originate mostly from D1R-MSN to D1R-MSN pairs (Taverna et al. 2008) and to a lesser extent from extrastriatal GABAergic interneurons (Melzer et al. 2017) and striatal interneurons (Taverna et al. 2008). However, of these cells only D1R-MSNs express Foxp2 (Fong et al. 2018). Thus, aberrant GAD67 expression levels can be related to changes in Foxp2 expression. This could either be through direct regulation of GAD67 expression by Foxp2 or by indirect effects of altered Foxp2 levels. Foxp2 is known to affect the development of striatal cells in primary cell culture (Vernes et al. 2011) and impaired D1R-MSN development in vivo may account for changes in protein expression, such as reduced GAD67 levels. We found that GAD67 expression was significantly increased around D1R-MSN somata in *Foxp2*^*S321X*/+^ mice compared to wild-type controls (Fig. 3b), whilst GAD67 was not changed around D2R-MSNs (Suppl Fig. 2). Furthermore, protein expression analysis by western blot in dissected striatum from juvenile *Foxp2*^+/+^ and *Foxp2*^*S321X*/+^ mice showed GAD67 expression to be increased (Fig. 3c).

To assess whether or not the increased GAD67 expression could be due to a general increase in expression of key components of GABA transmission, we quantified vesicular GABA transporter (VGAT) expression around D1R-MSN somata (Fig. 3a). No change in VGAT expression was observed between *Foxp2*^+/+^ and *Foxp2*^*S321X*/+^ mice, which suggests that Foxp2 specifically regulates GAD67 but does not affect the number of synapses. The increase in GAD67 levels of mice with reduced Foxp2 expression is consistent with the hypothesis that Foxp2 normally acts to repress the transcription of *Gad1* and is supported by the prior ChIP–chip data (Vernes et al. 2011). Differences in GAD67 expression have been described as a cause for changes in presynaptic GABA content and inhibitory activity (Lau and Murthy 2012). Thus, reduced Foxp2 expression could lead to increased inhibitory drive of D1R-MSNs through increased GABA production.

### Presynaptic GABA content is increased upon heterozygous loss of Foxp2 function

**Fig. 4:**
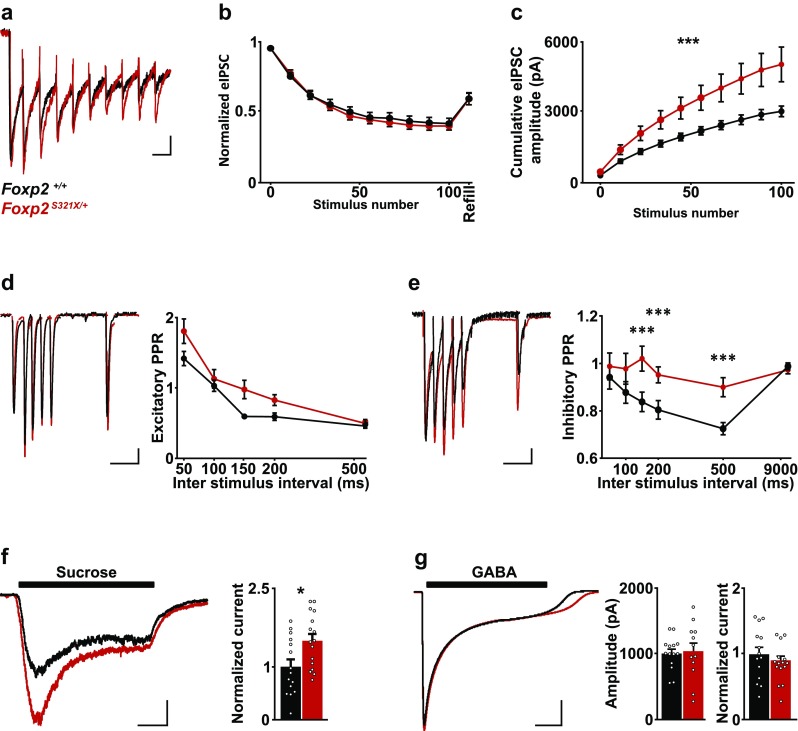
Presynaptic GABA content in juvenile (PND10-14) striatal D1R-MSNs is increased upon decreased Foxp2 expression. **a** Example traces of vesicle depletion following train stimulation (10 Hz, 100 stimuli) in *Foxp2*^+/+^ and *Foxp2*^*S321X*/+^ D1R-MSNs, every 10th response is shown. Scale 100 ms/50 pA. **b** Normalized (to first pulse) IPSC response during train stimulation. **c** Cumulative IPSC amplitude during train stimulation of D1R MSNs, (*Foxp2*^+/+^ = intercept 294.24 ± 85.3 pA, cumulative 3112.9 ± 286.4 pA, *Foxp2*^*S321X*/+^ = intercept 498.9 ± 301.3 pA, cumulative 5142.1 ± 484.6 pA, *P* < 0.001, two-sided Student’s *T* test). *N* number of mice, *n* number of cells. *Foxp2*^+/+^ *N*/*n* = 3/16, *Foxp2*^*S321X*/+^ *N*/*n* = 3/16. **d** Example trace of excitatory PPR at different inter stimulus intervals (ISI) in D1R-MSNs of *Foxp2*^+/+^ and *Foxp2*^*S321X*/+^ mice. Scale 100 ms/50 pA. Quantification of paired pulse ratio (PPR) (50, 100, 150, 200, 500ms: *Foxp2*^+/+^ 1.573 ± 0.08, 1.284 ± 0.06, 0.958 ± 0.02, 0.955 ± 0.04, 0.856 ± 0.02 vs *Foxp2*^*S321X*/+^ 1.864 ± 0.13, 1.357 ± 0.1, 1.244 ± 0.1, 1.131 ± 0.06, 0.884 ± 0.04, NS, Repeated measures ANOVA). *Foxp2*^+/+^ *N*/*n* = 2/8, *Foxp2*^*S321X*/+^ *N*/*n* = 3/11. **e** Same as **d** but for inhibitory PPR, (50, 100, 150, 200, 500 ms: *Foxp2*^+/+^ 0.936 ± 0.05, 0.872 ± 0.05, 0.832 ± 0.04, 0.798 ± 0.04, 0.718 ± 0.03 vs *Foxp2*^*S321X*/+^ 0.983 ± 0.06, 0.973 ± 0.07, 1.016 ± 0.05, 0.947 ± 0.03, 0.894 ± 0.04. *P* < 0.001 for 150, 200, 500 ms, repeated measures ANOVA). *Foxp2*^+/+^ *N*/*n* = 3/23, *Foxp2*^*S321X*/+^ *N*/*n* = 3/20. PPR is normalized to the first pulse. **f** Example trace of postsynaptic inhibitory response to forced vesicle exocytosis during 10 s local application of 500 mM sucrose. Scale 2 s/50 pA. Normalized (to wild-type) current transfer during 10 s sucrose application (*Foxp2*^+/+^ = 9.1 × 10^5^ ± 1.26 × 10^5^ pA, *Foxp2*^*S321X*/+^ = 13.4 × 10^5^ ± 1.6 × 10^5^ pA, *P* < 0.01, two-sided Students’ *T* test). *Foxp2*^+/+^ *N*/*n* = 2/15, *Foxp2*^*S321X*/+^ *N*/*n* = 3/16. **g** Example trace of postsynaptic response during 10 s local application of 100 µM GABA. Scale 2 s/200 pA. Quantification of peak amplitude and total current transfer during GABA application (*Foxp2*^+/+^ = 1.00 × 10^3^ ± 66.7 pA, *Foxp2*^*S321X*/+^ = 1.04 × 10^3^ ± 125 pA, NS, total current transfer *Foxp2*^+/+^ = 4.8 × 10^6^ ± 3.4 × 10^5^ pA, *Foxp2*^*S321X*/+^ = 5.4 × 10^6^ ± 5.5 × 10^5^ pA, NS, two-sided Student’s *T* test). *Foxp2*^+/+^ *N*/*n* = 2/14, Foxp2^S321X/+^ *N*/*n* = 2/12. *P < 0.05, ***P < 0.001

As GAD67 levels directly correlate with presynaptic GABA production, we explored if the increased GAD67 levels following reduced Foxp2 expression lead to elevated presynaptic GABA concentration. Presynaptic GABA is stored in vesicles, and is released upon electrical or pharmacological stimulation of the neuron (Alabi and Tsien 2012). A 10 s 10 Hz stimulation protocol has been described that efficiently depletes the entire readily releasable GABA vesicle pool (RRP) (Maas et al. 2017; Chen et al. 2017). This depletion protocol can be used to compare the quantal content of the GABA RRP between D1R-MSNs of wild-type and *Foxp2*^*S321X*/+^ mice. We show that this stimulation protocol indeed depletes the RRP in juvenile wild-type and *Foxp2*^*S321X*/+^ mice (Fig. 4a, b). However, in *Foxp2*^*S321X*/+^ mice the average current transferred per stimulation, as well as the cumulative current transferred after 100 stimulations, was significantly increased compared to wild-type controls (Fig. 4c). However, we did not observe a difference in the kinetics of release when release was normalized, which indicates that vesicle recycling was not affected in *Foxp2*^*S321X*/+^ mice (Fig. 4b).

Changes in GABA concentration at the synapse can affect synaptic strength and vesicle release probability (Olpe et al. 1994; Jensen et al. 1999). We, therefore, examined both excitatory and inhibitory paired pulse ratios (PPRs) in juvenile Foxp2^S321X/+^ mice and littermate controls. No differences in excitatory PPRs were found between genotypes (Fig. 4d). However, in contrast to the expected increase in inhibitory PPR Foxp2^S321X/+^ mice showed a lack of inhibitory paired pulse depression, specifically at longer inter stimulus intervals (Fig. 4e). The lack of inhibitory PPD can be explained increased GABA release per stimulation (Fig. 4a). If only a fraction of the total released GABA is necessary to saturate postsynaptic GABA, then reduction of vesicles released with subsequent stimulations would not lead to PPD, because enough GABA is still released to saturate the postsynaptic GABA receptors that are present.

Next, we sought to confirm the increased presynaptic GABA release pharmacologically, to exclude aberrant effects from recurrent stimulation. Local application of 500 mM sucrose for 10 s (Lipstein et al. 2017) efficiently induced vesicle exocytosis in juvenile *Foxp2*^+/+^ and *Foxp2*^*S321X*/+^ mice (Fig. 4f). The total current transfer during sucrose application was increased by approximately 50% in D1R-MSNs from *Foxp2*^*S321X*/+^ mice, similar to the increase in current transfer observed upon electrical stimulation (Fig. 4f).

Finally, the increase in mIPSC amplitude (Figs. 2b, 3h) in mice with reduced Foxp2 expression also suggests postsynaptic GABA receptor abundance might be increased. We used local application of GABA to investigate if postsynaptic GABA_A_ receptor presence was affected by reduced Foxp2 expression. GABA application elicited a strong response in D1R-MSNs of both *Foxp2*^+/+^ and *Foxp2*^*S321X*/+^ mice (Fig. 4g). No difference in peak response amplitude or total current transfer could be observed between genotypes (Fig. 4g). Taken together, our data suggest that D1R-MSNs exhibit increased GABA content at the presynapse following reduced Foxp2 expression, leading to a heightened quantal GABA release. This in turn leads to elevated inhibition of the striatal direct pathway.

### Pharmacological manipulation of inhibition partially rescues motor skill learning deficits in *Foxp2*^*S321X*/+^ mice

Because aberrant regulation of direct pathway inhibitory activity has been shown to produce motor skill learning deficits (Rothwell et al. 2014; Zhang et al. 2015), we next investigated whether blocking inhibitory activity might be an effective in vivo intervention. One of the most pronounced behavioral deficits displayed by mice with heterozygous mutations in *Foxp2* is decreased motor skill learning, shown by impaired performance on the accelerating rotarod (Groszer et al. 2008; French et al. 2012). Increased inhibition of the direct pathway as demonstrated herein could help explain why *Foxp2* mutations lead to impaired rotarod performance, since successful acquisition of this task is dependent on precise regulation of striatal activity. Cui et al. (2008) showed that increases in presynaptic GABA content cause learning and memory deficits when present in hippocampal neurons. Intriguingly, they found that learning and memory improved dramatically after a low concentration intraperitoneal (IP) injection with picrotoxin (PTX), a compound that blocks GABA_A_ receptor mediated inhibition (Cui et al. 2008). We, therefore, hypothesized that a low dose of PTX might be able to ameliorate the motor skill learning deficits present in the *Foxp2*^*S321X*/+^ mice in a similar manner.

**Fig. 5:**
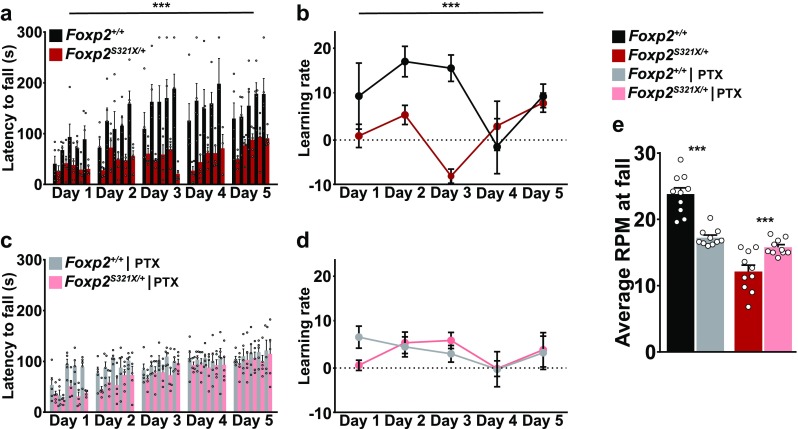
Pharmacological blockade of inhibition modulates rotarod performance and motor skill learning. **a** *Foxp2*^*S321X*/+^ mice show impaired motor skill learning, shown by the decreased latency to fall (in seconds) across training sessions (days 1–5: *Foxp2*^+/+^ 75.3 ± 9.7, 120.4 ± 14.4, 164.7 ± 13.6, 165.5 ± 12.2, 160.7 ± 10.8. *Foxp2*^*S321X*/+^ 34.4 ± 3.1, 52.5 ± 7.4, 53.1 ± 8.7, 55.0 ± 8.15, 82.6 ± 8.38. *P* < 0.01, repeated measured ANOVA). Each session consists of five trials of 5 min, during which the rotarod accelerated from 4 to 40 rpm. **b** Both *Foxp2*^+/+^ and *Foxp2*^*S321X*/+^ mice show a positive learning rate during most sessions, with *Foxp2*^+/+^ mice having a significantly higher learning rate (days 1–5: *Foxp2*^+/+^, 10 ± 7.5, 17.8 ± 3.5, 16.3 ± 3.00, − 1.4 ± 6.3, 10 ± 2.7. *Foxp2*^*S321X*/+^, 0.9 ± 2.7, 5.7 ± 2.2, − 8.2 ± 1.6, 3.2 ± 5.6, 8.5 ± 2.2. *P* < 0.05, repeated measures ANOVA), learning rate was calculated as: ${\text{Learning}}\;{\text{rate}}=\frac{{{\text{latency}}\;{\text{to}}\;{\text{fall}}~({\text{session}}\;~5 - {\text{session}}\;~1)}}{{{\text{number}}\;{\text{of}}\;{\text{trials}}}}$. **c, d** *Foxp2*^+/+^ mice subjected to 0.1 mg/kg intraperitoneal injection of PTX show decreased rotarod performance and learning rates, whereas these were increased in *Foxp2*^*S321X*/+^ mice (latency to fall: days 1–5: *Foxp2*^+/+^ 71.7 ± 12.4, 91.7 ± 5.5, 96.4 ± 4.7, 104.4 ± 1.2, 114 ± 3.2. *Foxp2*^*S321X*/+^ 33.8 ± 4.5, 59.8 ± 5.7, 77.4 ± 5.3, 91.8 ± 1.1, 103.2 ± 3.61. NS learning rate: *Foxp2*^+/+^ 7.2 ± 2.5, 5 ± 2.3, 3.4 ± 1.9, − 0.1 ± 4.0, 3.6 ± 3.8. *Foxp2*^*S321X*/+^, 0.76 ± 1.2, 5.9 ± 2.5, 6.4 ± 1.9, 0.0 ± 1.8, 4.3 ± 3.9. NS, repeated measures ANOVA). **e** Average RPM at which mice fail the accelerating rotarod task during session 4 and 5 in vehicle and PTX conditions (vehicle, *Foxp2*^+/+^ 24.1 ± 0.94 RPM, *Foxp2*^*S321X*/+^ 12 ± 0.96 RPM, *P* < 0.001. PTX, *Foxp2*^+/+^ 16.7 ± 0.4 RPM, *Foxp2*^*S321X*+^ 15.3 ± 0.372 RPM, NS, two-sided Students’ *T* test). For all treatment conditions, *N* = 5 mice. ***P < 0.001

We first validated the presence of motor skill learning deficits in our *Foxp2*^*S321X*/+^ mice by measuring their performance and learning rate on the accelerating rotarod during five consecutive training days and comparing them to littermate controls (Fig. 5a, b). The impaired rotarod performance in adult *Foxp2*^*S321X*/+^ mice that we observed is consistent with previous reports on *Foxp2* heterozygous mutant mice (French et al. 2012; Groszer et al. 2008). Next, we assessed a viable treatment dose. Injection of 1 mg/kg PTX produced grand mal seizures in both *Foxp2*^+/+^ and *Foxp2*^*S321X*/+^ mice, whereas both 0.01 mg/kg and 0.05 mg/kg did not have any effect on rotarod performance (Suppl. Figure 3). An intermediate dose of 0.1 mg/kg did not induce seizures, but had a notable negative effect on the rotarod performance of pre-trained wild-type mice (Suppl. Figure 4), whereas the rotarod performance of pre-trained heterozygous mice was not affected.

We, therefore, injected wild-type and *Foxp2*^*S321X*/+^ mice with 0.1 mg/kg PTX 10 min prior to each training session and subjected them to the same motor learning paradigm as the vehicle-injected (DMSO) mice. Interestingly, this 0.1 mg/kg PTX injection differentially affected rotarod performance of wild-type and *Foxp2*^*S321X*/+^ mice. Both wild-type and Foxp2^S321X/+^ mice still show an increase in performance and a positive learning rate during sessions (Fig. 5c, d). Treatment with PTX had a profound negative effect on rotarod performance in *Foxp2*^+/+^ mice, whilst in *Foxp2*^*S321X*/+^ mice, rotarod performance was significantly increased compared to mice without treatment. These opposite effects of PTX treatment resulted in a comparable performance of *Foxp2*^+/+^ and *Foxp2*^*S321X*/+^ mice when comparing average rotarod speed (RPM) at fall from the last two trials, (Fig. 5e) with PTX treatment. This shows that decreasing inhibitory activity might be a viable method to ameliorate motor deficits induced by decreased expression of Foxp2 and corroborates our data that in mice with reduced Foxp2 expression the E/I balance is shifted towards increased inhibition.

## Discussion

Mutations in *FoxP2* affect striatal circuitry both in human cases of speech/language disorder and in animal models of Foxp2 dysfunction (Liegeois et al. 2003; Schulz et al. 2010; Groszer et al. 2008; French et al. 2012). Here, we show that Foxp2 affects both excitatory and inhibitory striatal activity in a cell-specific manner during development and in adulthood. Foxp2 is predominantly expressed in striatal direct pathway D1R-MSNs. Decreased Foxp2 expression leads to reduced excitatory activity and increased inhibitory activity in D1R-MSNs. Molecular evidence suggests that the increase in inhibitory activity is due to a de-repression of GAD67 expression. The number of GAD67-positive puncta around the somata of D1R-MSNs increases when Foxp2 expression is reduced, which is accompanied by increased presynaptic GABA content and increased inhibition of the striatal direct pathway. Intriguingly, blocking inhibition with PTX results in a partial rescue of motor skill learning deficits in *Foxp2*^*S321X*/+^ mice, whereas wild-type littermates show impaired motor skill learning after treatment.

Striatal excitatory connections are formed exclusively by projections from external sources (Hunnicutt et al. 2016). Subpopulations of cortical and thalamic projection neurons form excitatory connections to the striatum (Pan et al. 2010; Hintiryan et al. 2016), and these brain regions contain Foxp2-positive cells as well (Lai et al. 2003; Takahashi et al. 2003; Vargha-Khadem et al. 2005; Hisaoka et al. 2010; Sia et al. 2013). However, it is currently unknown if the cortical and thalamic neurons that express Foxp2 project to the striatum. Our data show that reduced Foxp2 expression decreases D1R-MSN mEPSC amplitude, without influencing mEPSC frequency or excitatory PPR. This suggests that only postsynaptic excitatory strength is affected, and excitatory inputs to the striatum are not affected by reduced Foxp2 expression. Furthermore, the lack of excitatory presynaptic changes in striatal MSNs indicates that excitatory cortical and thalamic cells which do express Foxp2 either do not project to MSNs in the dorsolateral striatum or that Foxp2 has no presynaptic function in these neurons.

Concurrent with the decrease in excitatory activity, we observed an increase in inhibitory activity of striatal D1R-MSNs. Gene ontology analysis following Foxp2-ChIP experiments (Vernes et al. 2011), which groups significantly regulated genes among common biological pathways, has suggested GABA signaling pathways are regulated by Foxp2. Striatal MSNs express both GAD67 and GAD65, two catalytic enzymes involved in the production of GABA (Laprade and Soghomonian 1999). To our knowledge, GAD65 has not been identified as a regulatory target of Foxp2, and mRNA levels of *Gad2* (the gene which codes for GAD65) are unaltered when Foxp2 expression is reduced (French et al. 2007). In contrast, the *Gad1* gene is clearly a regulatory target of Foxp2, shown by ChIP (Vernes et al. 2011), and we show that expression of its protein product GAD67 is increased around striatal D1R-MSNs of *Foxp2*^*S321X*/+^ mice. The direct regulation of *Gad1* by Foxp2 suggests Foxp2 expression is necessary to regulate GAD67 levels in the striatum. However, we cannot rule out that GAD67 levels might be altered in striatal interneurons through non-cell autonomous mechanisms dependent on Foxp2. Interestingly, increased GAD67 expression levels have been causally linked to increased presynaptic GABA content (Chao et al. 2010; Hibbert et al. 2004) and enhanced GABA transmission (Krishnan et al. 2015), both of which occur in D1R-MSNs from *Foxp2*^*S321X*/+^ mice.

Spine formation and excitatory activity in striatal MSNs are affected in homozygous Foxp2 knockout mice during early postnatal development, possibly through increased Mef2C expression. Mef2C is a transcription factor, which acts as a developmental brake on glutamatergic synapse formation and is regulated by Foxp2 (Chen et al. 2016). However, we show that decreased excitatory activity in D1R-MSNs is present in both juvenile and adult *Foxp2*^*S321X*/+^ mice. Mef2C expression is virtually absent in adolescent mouse striatum (Chen et al. 2016). This suggests that the decrease in excitatory activity could be caused by impaired generation of glutamatergic synapses during early development, which can have lasting effects on physiology and behavior in adult mice (Harrington et al. 2016). Intriguingly, Mef2C, has been shown to regulate the activity of both excitatory and inhibitory synapses in cortex in a cell-autonomous way (Harrington et al. 2016). Knockout of *Mef2C* decreased excitation and increased inhibition in cortex, similar to the physiological changes that we show in striatal D1R-MSNs of *Foxp2*^*S321X*/+^ mice. Dysregulation of striatal Mef2C expression following heterozygous *Foxp2* loss of function could, therefore, be partially responsible for the striatal E/I imbalance that we measured.

Our findings show that reduced Foxp2 expression disrupts striatal E/I balance, which is dynamically regulated through pre-and postsynaptic mechanisms (Abbott and Nelson 2000; Bolshakov and Siegelbaum 1994; Bi and Poo 1998; Yang and Calakos 2013). Whilst the decreased excitatory activity seems to originate postsynaptically, our data suggest that reduced Foxp2 expression leads to increased presynaptic GABA production. D1R-MSNs form extensive connections toward the substantia nigra (SN), such that reduced activation of D1R-MSNs leads to reduced inhibition of the SN. Increased release of GABA could be a cell-autonomous mechanism to increase the inhibitory drive of D1R-MSN projections toward the SN. However, intra-striatal inhibition is governed by MSNs as well: D1R-MSNs project to other D1R-MSNs (Taverna et al. 2008). This means that a feedback loop could occur to increase inhibitory drive, which would result in stronger inhibition of striatal D1R-MSNs. Paired recording of striatal D1R-MSNs in *Foxp2*^+/+^ and *Foxp2*^*S321X*/+^ could help to determine whether such a feedback loop is present and if such a mechanism can negate the effect of the increased presynaptic GABA production in presynaptic terminals within the SN.

Furthermore, the striatal E/I imbalance following reduced Foxp2 expression is maintained throughout development and in adult mice. This can explain why impaired striatal plasticity and motor skill learning deficits are present in adult mice with heterozygous *Foxp2* mutations (French et al. 2012; Groszer et al. 2008). Interestingly, in a mouse model for neuroligin-3 (NL-3) dysfunction, known to produce similar behavioral and physiological phenotypes as mutation of *Foxp2*, adult re-expression of NL-3 rescues motor skill learning deficits (Rothwell et al. 2014). Restoration of E/I balance in adulthood could, therefore, be a viable strategy to ameliorate the motor learning deficits observed upon reduced Foxp2 expression. Modulation of GABAergic activity using GABA_A_ antagonists has been shown to improve learning and memory in mouse models for cognitive disorders (Rueda et al. 2008; Cui et al. 2008) and phase I clinical trials are underway to test GABA_A_ antagonists on people with Down syndrome (Contestabile et al. 2017). We show that modulation of GABAergic activity by partially blocking inhibitory activity increases motor skill learning in *Foxp2*^*S321X*/+^ mice. Intriguingly, wild-type mice were adversely affected by the same PTX treatment, which indicates that successful modulation of GABAergic activity might be highly dose dependent.

Taken together, we show for the first time that reduced Foxp2 expression bidirectionally affects both excitatory and inhibitory activity of striatal direct pathway MSNs, throughout development as well as in adult mice. Partially blocking inhibitory activity in vivo might restore this E/I imbalance, and we found that this intervention had a positive effect on motor skill learning in mice with reduced Foxp2 expression. Restoring the E/I balance by pharmacologically modulating inhibitory activity might be a feasible therapeutic intervention for complex motor disorders.

### Materials and methods

#### Mouse lines

The experimental procedures were approved by the Animal Ethics Committee of the Radboud University Nijmegen, under DEC application number 2014-098 (Nijmegen, The Netherlands) and conducted in accordance with the Dutch legislation. Every effort was made to minimize animal discomfort and the number of animals used.

The Foxp2-S321X line was maintained on a C57BL/6J background, and heterozygotes and wildtype littermates between PND11 and PND17 (juvenile) or PND55-65 (adult) were used for the immunofluorescent stainings and electrophysiological recordings. The generation, marker-assisted backcrossing and genotyping of this strain are fully described in (Groszer et al. 2008; Keays et al. 2006; Coghill et al. 2002). BACtrap mice carrying GFP under the D1R promoter (D1R-GFP) or D2R promoter (D2R-GFP) were originally generated by the GENSAT (Gene Expression Nervous System Atlas) (Gong et al. 2003) and backcrossed to C57BL6/J mice.

### Electrophysiology

Experiments were conducted on 350 µm thick coronal slices. Mice (PND11-17 or PND55-65) were sacrificed by decapitation following isoflurane anesthesia. Slices were cut using a vibratome (HM650V Thermo Scientific) in cooled (4 °C) artificial cerebrospinal fluid containing (in mM): 87 NaCl, 11 Glucose, 75 Sucrose, 2.5 KCl, 1.25 NaH_2_PO_4_, 0.5 CaCl_2_, 7 MgCl_2_, 26 NaHCO_3_, continuously oxygenated with 95% O_2_/5% CO_2_. Collection of slices started when the striatum became visible and slices were collected until the hippocampus was visible. After collection, slices were incubated at 32 °C in oxygenated ACSF for at least 1 h before recording. Slices were transferred to the recording setup 10 min prior to recording and incubated in recording ACSF containing (in mM): 124 NaCl, 3 KCl, 1.25 NaH_2_PO_4_, 2 CaCl_2_, 1 MgCl_2_, 26 NaHCO_3_, 10 Glucose, continuously oxygenated and heated to 32 °C. Patch pipettes (3.5–5.5 MΩ) were made from borosilicate glass capillaries and filled with intracellular solution containing: 115 CsMeSO_3_; 10 CsCl; 10 HEPES; 2.5 MgCl_2_; 4 Na2ATP; 0.4 NaGTP; 10 Na-Phosphocreatine; 0.6 EGTA, 10 QX-314. Activity was recorded using a Digidata 1440A digitizer and a Multiclamp 700B amplifier (Molecular Devices). Sampling rate was set at 20 kHz and a lowpass 1 kHz filter was used during recording. All recordings were conducted in the dorsolateral quadrant of the striatum.

#### Miniature postsynaptic currents

mEPSCs were recorded in the prescience of Tetrodotoxin (TTX, 1 µM, Tocris) and Picrotoxin (PTX, 100 µM, Tocris) at a holding voltage of − 60 mV. mIPSCs were recorded in the presence of Tetrodotoxin (TTX, 1 µM, Tocris), 6-cyano-7-nitroquinoxaline-2,3-dione (CNQX, 5 µM, Tocris) and (2R)-amino-5-phosphonovaleric acid (APV, 100 µM, Tocris) at a holding voltage of + 10 mV.

#### GABA/AMPA ratio

All stimulation experiments were conducted by stimulation of afferent corticostriatal and intrastriatal axons using a bipolar concentric stimulus electrode (FHC, Bowdoin, Maine) placed in the dorsolateral striatum. GABA/AMPA ratio was measured in the presence of APV (100 µM). Cells were voltage-clamped at − 60 mV and a 1 ms stimulus from a bipolar tungsten electrode was given to record the AMPA response. Subsequently cells were clamped at 0 mV and the GABA response was measured.

#### Paired pulse ratio

Excitatory PPR was measured in the presence of PTX (100 µM) and APV (100 µM) with voltage clamped at − 60 mV. Inhibitory PPR was measured in the presence of CNQX (5 µM) and APV (100 µM) with voltage clamped at − 60 mV. Stimulation strength was set to evoke an approximately 200 pA response to the first stimulus. Two 1 ms pulses were given with a 50 ms, 100 ms, 150 ms, 200 ms, 500 ms or 9000 ms (inhibitory PPR only) interval. PPR was calculated as the peak 2/peak 1 ratio after correcting for any residual current at the second pulse.

#### GABA vesicle depletion

One millisecond pulses were given at 10 Hz for 10 s to entirely deplete the presynaptic GABA vesicle pool. After each 10 s stimulus train, cells were given 0.2 Hz stimulations for 40 s to assess the recovery of the vesicle pool between each stimulus train. One recording consisted of 10 consecutive stimulus trains. Cells were recorded in the presence of CNQX and at a holding voltage of − 60 mV.

#### Compound application

Sucrose (500 mM) or GABA (20 µM) was applied using a pressure ejection system (PDES-2DX, NPI, Tamm, Germany). The injection pressure was set to 5 psi/0.4 bar and injection duration was set to 10 s. Interinjection interval was set to 1 min. Compounds were delivered using a micropipette positioned at 30 µm from the target cell soma.

### Immunofluorescence

Animals were sacrificed by decapitation and whole mouse brain was fixed in 4% paraformaldehyde (PFA)/4% sucrose for 24 h. 60 µm coronal sections including the striatum were cut using a vibratome (Leica VT1000S, Leica microsystems). Slices were transferred to 1x Phosphate buffered saline (PBS) for immunofluorescent staining. The following antibodies were used: FoxP2 (Santa Cruz Sc-21069, 1:500), GAD67 (Millipore MAB5406, 1:200). Imaging was done using a Zeiss upright fluorescent microscope with apotome (Zeiss Axio Images, Oberkochen, Germany) using a 63 × oil immersion objective. For subsequent analysis of immunofluorescent staining, at least four slices per animal were analyzed. Data were normalized and average values for each animal were taken as the independent variable for further statistical analysis. Images were analyzed offline using FIJI (Fiji is just imageJ) image analysis software.

### Intraperitoneal injection

$Foxp{2^{S321X/+}}$ mice and wild-type littermate controls were injected intraperitoneally with either vehicle (DMSO) or 0.1 mg/kg picrotoxin (Tocris, Bristol, UK). Injection was done by hand and mice were placed back in their home cage for 10 min following injection, after which mice were placed on the accelerating rotarod.

### Accelerating rotarod

$Foxp{2^{S321X/+}}$ mice (6–8 weeks old) and wild-type littermate controls were placed on an accelerating rotarod (LE8200, Harvard apparatus) which increased rotation speed from 4 to 40 r.p.m. over a 5-min period. Mice were trained for five consecutive days, with five trials per day. Latency to fall (in seconds or RPM at fall) was scored, and mice were placed back in their home cage for 5 min between trials.

### Western blot

PND10-15 *Foxp2*^*S321X*/+^ animals and wild-type controls were sacrificed by decapitation. The striatum was dissected from separated hemispheres, frozen in liquid nitrogen and kept at − 80 °C. Samples were homogenized in 200 µl of lysis buffer (50 mM Hepes pH 7.4, 140 mM NaCl, 0.1% Triton-X100, 1% Tween 20, 0.1% deoxycholate) containing protease inhibitor mix (Roche Diagnostics). Protein levels were assessed using BCA. Sodium dodecyl sulfate polyacrylamide gel electrophoresis (SDS-PAGE) on 10% (w/v) at 200V for 30 min was carried out using a Mini-Protean system (Bio-Rad, USA). Protein (50 µg) was loaded in each lane with loading buffer (0.25 M Tris–HCl, pH 6.8, 2% SDS, 10% glycerol, 0.25% bromophenolblue, 4% beta-mercaptoethanol). After electrophoresis, proteins were transferred to a polyvinylidene fluoride membrane (PVDF, Amersham, Hybond-P), using an electrophoretic transfer system (Bio-Rad, USA). The membranes were then blocked with 5% skimmed milk dissolved in TBS-tween 0.1% for 1 h. The membranes were incubated overnight at 4 °C with the primary antibodies diluted in blocking buffer containing 1% skimmed milk dissolved in a TBS-Tween. The primary antibodies were the following: mouse monoclonal anti-bodies GAD67 (1:1000, Abcam), and GAPDH as a control (1:1000, cell signaling). After being washed for 1 h with 1% skimmed milk in TBS-T (0.05%), the membranes were incubated for 1 h in the dark at room temperature with goat-anti-mouse secondary antibody (1:5000; Bio-Rad, Goat-anti-mouse HRP conjugated). The membranes were imaged using a Chemidoc Touch imaging system (Bio-rad, Hercules, CA) and the generated pictures were quantified using ImageJ software. The levels of protein expression were normalized to GAPDH. Protein expression values are normalized to *Foxp2*^+/+^ expression (relative intensity).

### Statistics

Sample size was calculated assuming power of 0.8 and effect size *d* = 0.8, data are acquired from at least three mice for each genotype. All data are shown as mean ± SEM. Analysis between two groups was done using Students’ *T* test when normally distributed, or Mann–Whitney *U* rank-sum analysis when data did not pass normality. Analysis between multiple groups using repeated measures ANOVA. All statistical analysis was conducted in PRISM (Graphpad PRISM 7.00, Graphpad Software, San Diego, CA).

## Electronic supplementary material

Below is the link to the electronic supplementary material.

Supplementary material 1 (DOCX 306 KB)
