# Supplementary material for: Foxp2 loss of function increases striatal direct pathway inhibition via increased GABA release
Source: Brain Struct Funct. 2018 Sep 5;223(9):4211–26. doi: 10.1007/s00429-018-1746-6 (PMC6267273; doi:10.1007/s00429-018-1746-6)
Supplement: Supplementary file 1 — Supplementary material 1 (DOCX 306 KB) [file 429_2018_1746_MOESM1_ESM.docx]

**Supplementary Figures**

**Title:** Heterozygous Foxp2 loss of function increases striatal direct pathway inhibition via increased GABA release **Journal:** Brain structure and function

**Authors:** Jon-Ruben van Rhijn, Simon E Fisher, Sonja C Vernes and Nael Nadif Kasri

**Corresponding author:** Nael Nadif Kasri

**Affiliations corresponding author:**

Department of Cognitive Neuroscience, Radboudumc, Donders Institute for Brain, Cognition and Behaviour, 6525HR, Nijmegen, the Netherlands

Department of Human Genetics, Radboudumc, Donders Institute for Brain, Cognition, and Behaviour, 6525GA, Nijmegen, the Netherlands


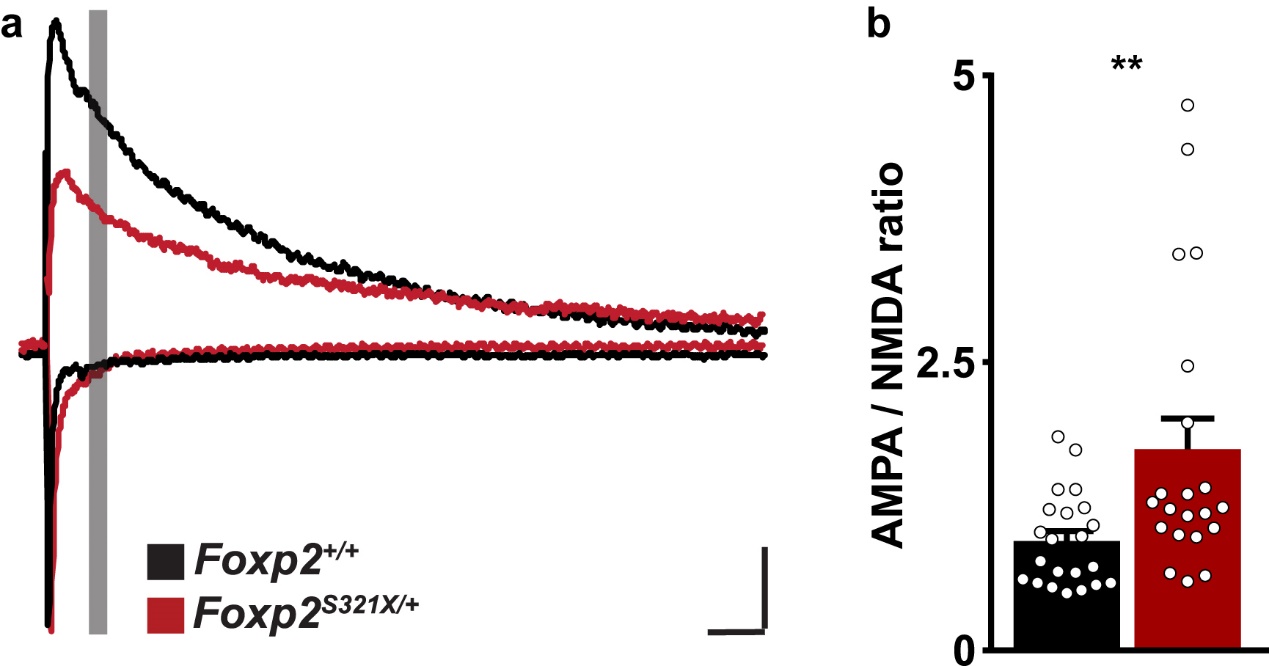


**Supplementary Figure 1 AMPA/NMDA ratio is increased in Foxp2S321X/+ juvenile mice (PND11-13).** (a) Example traces of AMPA/NMDA measurement by stimulation at -60mV (AMPA) and +40mV (NMDA) in the presence of PTX to block GABAergic neurotransmisstion. The AMPA response was mesaured as the peak response, whereas the NMDA response was measured as the average (pA/ms) of the area between 60-65ms after the AMPA peak (grey shaded area). (b) AMPA/NMDA ratio (*Foxp2^+/+^* 0.94±0.09 N/n = 3/22*, Foxp2^S321X/+^* 1.75±0.27 N/n = 3/22). Scalebar = 100ms/50pA, ** = P<0.01. All data is reported as mean ± SEM. N/n = animals/cells


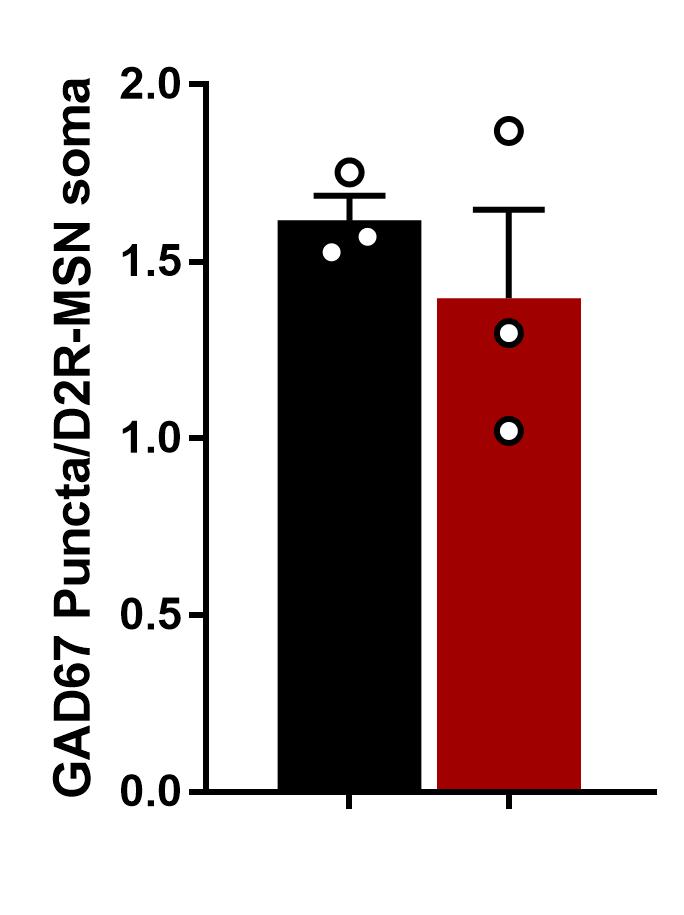


**Supplementary Figure 2: The number of GAD67 puncta surrounding D2R-MSN somata is not changed.** We counted GAD67 positive puncta surrounding D1R-negative (putative D2R) MSNs. No differences between the number of GAD67 puncta per soma were found between *Foxp2^+/+^* or *Foxp2^S321X/+^* mice. (*Foxp2^+/+^* = 1.6±0.07 puncta/D2R-MSN soma**, *Foxp2^S321X/+^* 1.4±0.25 puncta/D2R-MSN soma, NS, Mann-Whitney U.** ***Foxp2^+/+^* N/n = 3/12*, Foxp2^S321X/+^* N = 3/12. N/n = number of mice / number of slices**


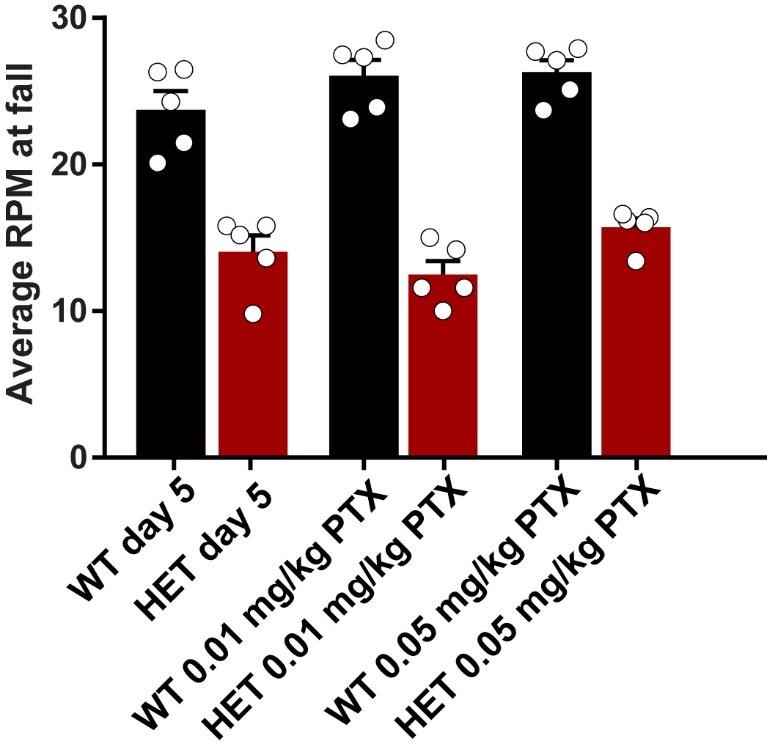


**Supplementary Figure 3. Low dose (0.01 or 0.05 mg/kg) IP PTX injection** **does not affect rotarod performance of *Foxp2^+/+^* or *Foxp2^S321X+^* mice.** The last trial day (day 5) and 2 subsequent days (0.01mg/kg injection or 0.05 mg/kg) are shown. Average RPM at fall is not affected by injection and not different between experimental days (*Foxp2^+/+^* 23.6±1.28, 25.9±2.4, 26.2±0.81 N.S. *Foxp2^S321X/+^* 14±1.13, 12.5±0.92, 15.7±0.59, NS, 2 Factor ANOVA, factors experimental day and PTX treatment). N = 5.


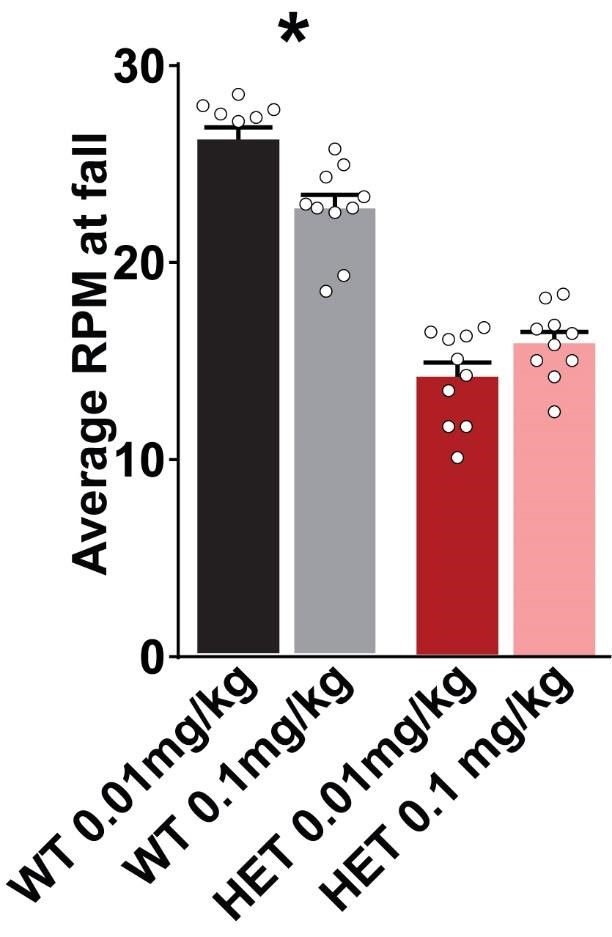


**Supplementary Figure 4. Pre-trained *Foxp2^S321X/+^* mice are not affected by PTX injection**. Average RPM at fall for pre-trained mice injected with either 0.01mg/kg PTX or 0.1mg/kg PTX. (*Foxp2^+/+^* 25.9±0.63, 23.2±0.71, P<0.05, *Foxp2^S321X/+^* 12.5±0.75, 14.04±0.58, NS, two-sided students T-test). N = 5.
